# Public Interest in Dry Eye Disease and Its Association With Environmental Parameters in Taiwan: Google Trends Infodemiology Study

**DOI:** 10.2196/74317

**Published:** 2025-11-14

**Authors:** Po-Chun Chang, Tsung-Hsien Tsai, Shih-Chieh Shao, Chi-Chin Sun

**Affiliations:** 1 Department of Medical Education, Chang Gung Memorial Hospital, Keelung, Taiwan; 2 Department of Ophthalmology, Chang Gung Memorial Hospital, No. 222, Maijin Road, Anle District, Keelung, 204201, Taiwan, 886 2-24313131, 886 2-24311190; 3 School of Medicine, College of Medicine, Chang Gung University, Taoyuan, Taiwan; 4 Department of Pharmacy, Chang Gung Memorial Hospital, Keelung, Taiwan; 5 School of Pharmacy, Institute of Clinical Pharmacy and Pharmaceutical Sciences, College of Medicine, National Cheng Kung University, Tainan, Taiwan

**Keywords:** dry eye disease, Google Trends, infodemiology, Taiwan, environmental parameters, public interest

## Abstract

**Background:**

A high prevalence of dry eye disease (DED) has intensified public health concerns in Taiwan. With the growing reliance on online resources for health information, platforms such as Google Trends (GT) provide a valuable method for capturing public interest. This approach also allows for the exploration of potential associations between public interest in DED and environmental parameters, which may further elucidate underlying factors contributing to the disease’s rising prevalence.

**Objective:**

This study aims to (1) analyze public interest in DED in Taiwan using GT data, (2) investigate correlations between search interest and environmental parameters, and (3) identify shifts in the focus of search over time.

**Methods:**

We analyzed GT data from December 2018 to July 2024, focusing on relative search volume (RSV) for DED across Taiwan and its 6 special municipalities. Temporal trends in RSV were assessed using spline regression models, and monthly variations were assessed using the Kruskal-Wallis test. The Spearman correlation analysis was used to evaluate the association between RSV and environmental parameters, while dynamic time warping analysis clarified the temporal alignment of RSV with these parameters. Rising search queries were analyzed to identify shifts in public interest over time. Furthermore, top Google search results for DED-related keywords were assessed for topic coverage, quality, and readability.

**Results:**

A significant rising trend in RSV for DED was observed over the study period in Taiwan (mean instantaneous derivative=0.445; *P*<.001) and across all 6 special municipalities. Environmental parameters such as methane (CH_4_), total hydrocarbons, and nonmethane hydrocarbons were identified as novel pollutants strongly correlated with RSV (*P*<.001), along with known pollutants such as nitric oxide (NO), nitrogen dioxide (NO_2_), sulfur dioxide (SO_2_), nitrogen oxides (NOx), and carbon monoxide (CO). Dynamic time warping analysis revealed the strongest temporal alignment was between RSV and hydrocarbons, including CH_4_ and total hydrocarbons, further emphasizing their potential role in influencing public interest. Assessment of web-based DED information of 80 websites revealed generally low quality (DISCERN score: mean 2.14, SD 0.40), and the average readability corresponded to a college reading level (grade: mean 21.1, SD 4.5). Rising search queries shifted from diagnostic and treatment methods before the COVID-19 pandemic to natural remedies during the COVID-19 lockdown and self-diagnosis and treatment options after the pandemic. Gaps were also identified between public interest and the availability of online information.

**Conclusions:**

Public interest in DED has increased significantly in Taiwan from 2018 to 2024, with hydrocarbons identified as strongly associated environmental parameters. The shifts in related queries reflect changing public interest, accentuating the need for health care information that aligns with public interest and addresses gaps in available resources.

## Introduction

### Background

Dry eye disease (DED) is a multifactorial ocular surface disease, with symptoms ranging from discomfort to significant visual impairment [1]. The prevalence of DED ranges from 5% to 50% globally and appeared to be higher in Asian populations than in White populations [2,3]. In a recent meta-analysis, the prevalence and incidence of DED in Asia were estimated to be 20.1% and 16.7%, respectively [4]. High prevalence of DED leads to substantial economic and human burdens, both worldwide and in Asia. In Taiwan, 33.7% of patients older than 65 years are estimated to have DED, and the incidence rates of DED increased at least 2-fold from 2001 to 2015, indicating the growing burden of the disease [5,6].

Despite the growing burden of DED, understanding the patterns of public awareness and information-seeking behavior is crucial for tracking the epidemiological pattern of a disease as well as improving patient education and care strategies [7]. With the rise of internet use, people increasingly rely on online resources for health information. Thus, infodemiology research allows researchers to track disease awareness and behavioral trends at a population level. By analyzing search patterns, we can capture the public’s real-time interest, helping to identify gaps between public knowledge and clinical understanding [8]. Google Trends (GT), a tool that tracks real-time search queries, has emerged as a valuable platform for analyzing public interest in ocular or environmental health conditions [9-11].

Prior GT studies mapped DED interest across US regions and explored its seasonal variations in Saudi Arabia [10,12], while broader environmental-health work has linked spikes in searches for symptoms such as cough or asthma to particulate matter with diameter ≤2.5 μm (PM₂.₅) and nitrogen dioxide (NO₂) levels [13-15]. To date, however, no study has examined how public interest in DED interacts with the full spectrum of local environmental factors, creating a research gap at the intersection of these fields.

### Objectives

Therefore, we aim to explore the public interest in DED in Taiwan using GT data in this study. Specifically, we seek to analyze temporal and seasonal trends, investigate the correlation between search patterns and environmental parameters, and assess the rising search queries related to DED. Furthermore, we will evaluate whether the information provided by web-based resources aligns with public interests as reflected in the search data.

## Methods

### Data Collection: GT

Our data were sourced from GT [16], which provides relative search volume (RSV)—a metric that reflects the level of search interest relative to the total number of search queries within a specific time frame and geographic location. RSV values are normalized between 0 and 100, enabling straightforward comparisons of search interest over time.

A unique feature of GT is its use of “topics,” which groups related searches across various languages and search term variations. For instance, a search for *dry eye* will return results under the *DED* topic, encompassing all searches linked to the condition, regardless of how users phrase their queries. In addition, the “rising queries” feature emphasizes search terms that have seen a substantial increase in volume over a specified time frame compared to the previous period, making it particularly useful for identifying emerging trends in public interest.

We initially identified global topics related to DED from 2004 onward, with 2 key topics emerging: *DED* and *dry eye*. Upon comparison, Taiwan exhibited the highest RSV for the *DED* topic worldwide, indicating a significant public interest in the condition within the region. Consequently, we focused our analysis on the *DED* topic for this study.

For the query analysis, we retrieved recent GT data spanning from December 2018 to July 2024. The COVID-19 lockdown period was defined based on Taiwan’s nationwide level 3 alert, which lasted from May 19, 2021, to February 28, 2022, and involved strict restrictions on public movement and social gatherings. The post–COVID-19 period spanned from March 2022 to July 2024, corresponding to the end of our data collection. To ensure a balanced comparison, the pre–COVID-19 period was defined as December 2018 to April 2021, matching the duration of the post–COVID-19 period. In addition, we used the rising queries feature in GT to analyze public interest in DED across these periods. We extracted all the top rising queries reported by GT for each defined period (pre–COVID-19, COVID-19 lockdown, and post–COVID-19) to capture the most prominent emerging interests. The rising queries in each period were identified and compared to assess changes in public interest toward DED throughout the COVID-19 pandemic. Rising related queries were then categorized into 5 groups based on their theme: symptoms, treatment, etiology or related diseases, diagnosis, and others.

### Data Collection: Environmental Parameters in Taiwan

We collected environmental and climate data from 6 special municipalities in Taiwan (Taipei City, New Taipei City, Taoyuan City, Taichung City, Tainan City, and Kaohsiung City) for the same period as the GT data (December 2018 to July 2024). The data were downloaded from the official websites of 2 primary agencies: the Central Weather Administration (CWA) of Taiwan and the Environmental Information Open Platform of Taiwan’s Ministry of Environment. Our analysis examined important meteorological parameters, including average temperature, average relative humidity, evaporation, global solar radiation, precipitation, days with precipitation, average wind speed, and average wind direction, which may affect the severity of DED [17]. We also included established pollutants previously linked to DED, such as PM₂.₅, NO₂, sulfur dioxide (SO₂), ozone (O₃), and carbon monoxide (CO) [18-21]. Furthermore, other routinely monitored major air pollutants from the CWA and Taiwan’s Ministry of Environment, including hydrocarbons such as methane (CH₄), total hydrocarbons (THC), and nonmethane hydrocarbons (NMHC), were incorporated into our analysis. These hydrocarbons are also recognized for their irritant properties, and previous studies suggested potential pathways for ocular surface impact, including direct and indirect injury to the corneal epithelium and conjunctiva [22,23]. The data collection process involved downloading monthly reports from the official websites of relevant agencies. Meteorological data were retrieved from the CWA. Atmospheric pollutant data were downloaded from the Environmental Information Open Platform of the Ministry of Environment of Taiwan. Monthly averages for each environmental parameter were aligned with the corresponding monthly RSV data for each city. A direct comparison was then made between environmental parameters and public search interest.

Falsification outcomes were commonly used to evaluate the likelihood of false-positive results in main study outcomes that might arise from unmeasured confounders or limitations in internal validity [24]. Public interest in glaucoma, a common ocular disease, was considered a falsification outcome in our GT analysis.

### Ethical Considerations

Our study used publicly available and anonymized data from GT and official government environmental databases in Taiwan (CWA and the Environmental Information Open Platform). As the data sources contained no identifiable private information, this research protocol was reviewed by the institutional review board of Chang Gung Memorial Hospital, which waived the requirement for formal approval and informed consent.

### Statistical Analysis

All statistical analyses were conducted using RStudio (version 4.3.0; R Foundation for Statistical Computing). *P*<.05 was considered statistically significant, and all primary *t* tests were 2-tailed. To evaluate temporal trends in RSV, we used spline regression models to capture potential nonlinear patterns over time. Specifically, a linear model was fitted with RSV as the response variable and a B-spline basis with 4 *df* of the numeric time variable as the predictor to flexibly model the trend. The first derivative of this fitted spline was computed at each monthly time point, representing the instantaneous rate of change in RSV per year. The mean instantaneous derivative (MID) was then calculated by averaging these instantaneous yearly rates and subsequently dividing by 12 to express the final rate as an average change in RSV units per month. A positive MID indicates an overall increasing trend in search interest, while a negative MID indicates a decreasing trend. A residual bootstrap (1000 iterations) provided 95% CIs and *P* values for MID. Monthly variations in RSV were examined using the Kruskal-Wallis test, and RSV data were grouped by month for Taiwan and its 6 special municipalities. The results were visualized using box plots to illustrate the distribution of RSV across different time frames.

The Spearman correlation was used to assess the association between RSV for DED and environmental parameters. Monthly GT RSV data were complete for the study period. Environmental data were averaged monthly. Before these analyses and the subsequent dynamic time warping (DTW), environmental data also underwent linear interpolation to address missing values, and both the RSV and environmental time series were standardized. Most monthly environmental data were complete across the 6 municipalities, except for Tainan. For Tainan, some climate parameters for September 2022 were missing and were imputed using linear interpolation. Additionally, the evaporation parameter was unavailable for Tainan and was thus excluded from the analyses for this municipality. The analysis was then conducted for each environmental parameter and RSV data across Taiwan and its 6 special municipalities, with significant correlations (*P*<.05) indicating a potential influence of the corresponding environmental parameters on public interest. No additional smoothing was applied to the monthly averaged environmental data series beyond the inherent effect of monthly averaging.

To more comprehensively account for potential nonlinear alignments or variable lags that might reflect delayed health impacts, DTW was used to evaluate the temporal alignment between RSV and multiple environmental parameters across 6 special municipalities in Taiwan. DTW is an algorithm that measures the similarity between 2 temporal sequences. It finds the optimal nonlinear alignment between the sequences, and the resulting DTW distance quantifies their dissimilarity.

To assess the alignment between topics of public interest and availability of public information, we searched Taiwan’s Google website using Chinese keywords related to DED, including 乾眼 (dry eye), 眼睛乾澀 (dry eye discomfort), 乾眼症 (DED), 眼淚分泌減少 (decreased tear secretion), 淚液分泌不足 (insufficient tear secretion), 眼睛乾燥 (dryness of the eyes), and the terms 眼睛乾 and 眼乾 (collectively, ocular dryness). For each keyword, the top 20 search results were collected, resulting in an initial dataset comprising a total of 160 websites. Duplicate websites, including those with identical URLs or substantially overlapping content from the same author or organization, were identified and removed. The content of these remaining websites was reviewed and compared against the rising queries from GT. We examined which topics from the rising queries were covered in the sampled websites, how frequently they appeared, and which were absent. Furthermore, the quality and readability of these websites were assessed. Quality was evaluated independently by 2 ophthalmologists using the 16-item DISCERN instrument, with each item scored from 1 to 5 [25]. Discrepancies in DISCERN scores were resolved through discussion and consensus between 2 ophthalmologists. Readability was assessed using a web-based tool [26]. The frequency of each related term that appeared in both the websites and rising queries was recorded. The detailed methodology and key findings of the study, including public interest trend analysis, environmental associations, shifts in public search behavior, and the web-based information assessment, are summarized in Table S1 in Multimedia Appendix 1.

## Results

### Trends in RSV for DED From 2018 to 2024 in Taiwan and 6 Special Municipalities: Temporal and Monthly Variation

**Figure 1.**
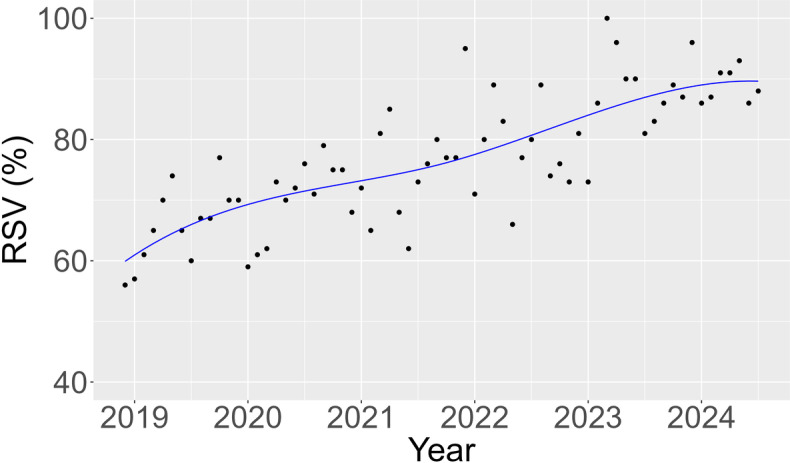
Trends in relative search volume (RSV) for dry eye disease in Taiwan from December 2018 to July 2024, based on Google Trends data. The blue line represents the overall RSV trend in Taiwan, with a mean instantaneous derivative computed at 0.445 per month (95% CI 0.308-0.593; *P* <.001), indicating a significant positive trend in public interest.

**Figure 2.**
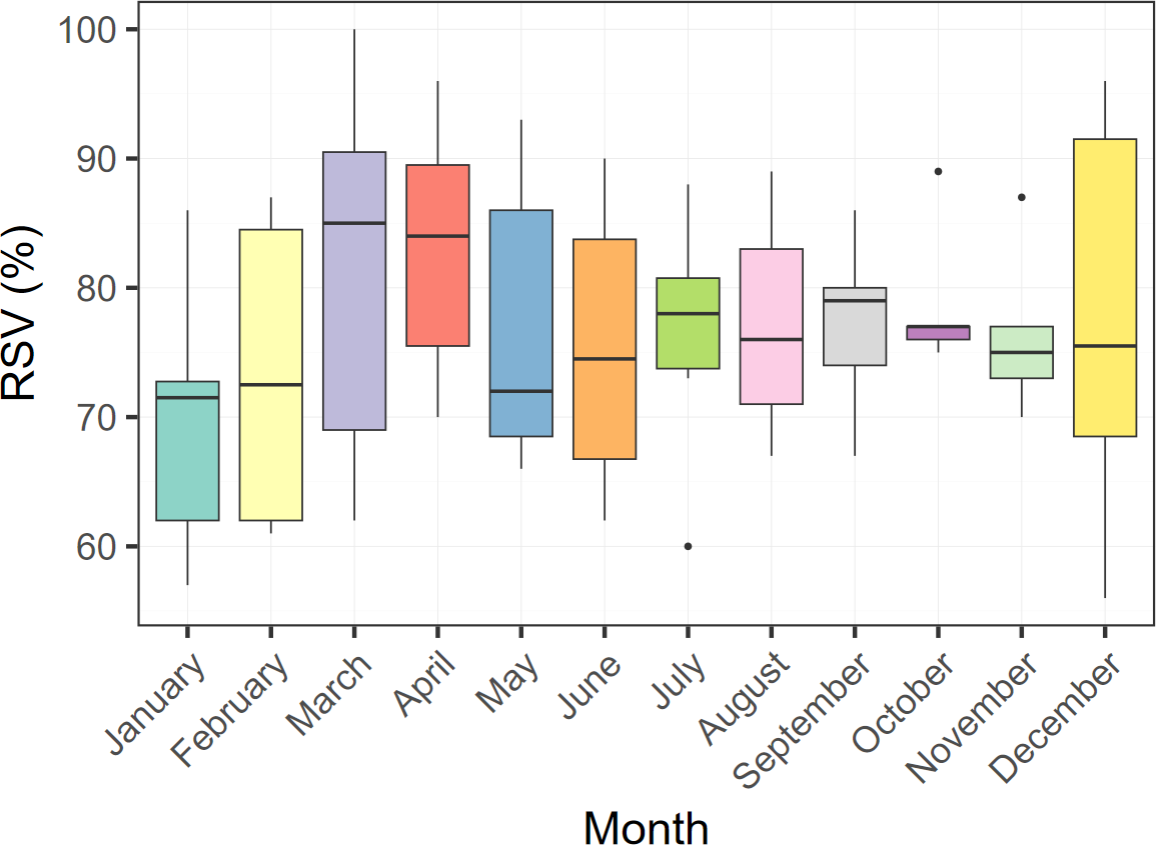
Monthly variations in relative search volume (RSV) for dry eye disease in Taiwan from December 2018 to July 2024. The Kruskal-Wallis test showed no statistically significant monthly differences in RSV (*P*=.85), suggesting consistent public search interest throughout the year.

Figure 1 presents the trends in RSV for DED from December 2018 to July 2024 in Taiwan. The spline regression analysis for Taiwan showed an adjusted *R*² of 0.619 with *P*<.001, as detailed in Figure 1 and Table S2 in Multimedia Appendix 1. Spline regression models for each city demonstrated significant positive trends in RSV over time, which is illustrated in Figure S1 in Multimedia Appendix 1. A geospatial visualization of these regional trends is provided in Figure S2 in Multimedia Appendix 1. The adjusted *R*² values varied among cities, with Taipei City showing the highest (adjusted *R*²=0.598; *P*<.001) and Taoyuan City the lowest (adjusted *R*²=0.228; *P*<.001). Significant positive trends in RSV were also observed in most cities. As a falsification outcome, glaucoma demonstrated no statistically significant trend over the study period (MID=0.120; *P*=.12; Figure S3 in Multimedia Appendix 1). Analysis for Taiwan also revealed no significant monthly differences (Figure 2).

### Correlation Between RSV and Environmental Parameters

The results of the Spearman correlation analysis revealed significant associations between RSV for DED and multiple environmental parameters across Taiwan and its 6 special municipalities. In Taipei (Table 1), air pollutants such as CH_4_, THC, SO_2_, CO, NO, and NMHC demonstrated the strongest significant correlation with RSV (*P*<.001), followed by NO₂, NOx, and average wind speed (*P*<.05). For other special municipalities across Taiwan, similar trends were also observed. CH_4_, SO_2_, and NMHC were significantly correlated to RSV in at least 5 of the 6 special municipalities (Table S3 in Multimedia Appendix 1). The results emphasized an association between air quality and public interest in DED. DTW analysis was used to further assess the temporal alignment between RSV and the environmental parameters identified as statistically significant in the Spearman correlation analysis. Across Taiwan’s 6 special municipalities, the resulting DTW distances revealed that air pollutants were among the most strongly correlated parameters. Consistently, hydrocarbons such as CH_4_ and THC, along with environmental parameters like ADT and AT, showed the smallest DTW distances, indicating the strongest temporal alignment with public search interest. A full list of DTW distances is available in Supplementary Table 4 in Multimedia Appendix 1.

**Table 1.** Spearman correlation between relative search volume (RSV) and environmental parameters across Taipei.

Parameter	Spearman correlation coefficient
Meteorological parameter	
AT^a^	0.032
ADT^b^	0.082
Evaporation	0.107
GSR^c^	0.206
Precipitation	−0.038
DP^d^	–0.191
ACC^e^	–0.071
AWS^f^	–0.315^g^
ARH^h^	0.134
AWD^i^	0.123
Pollutant	
PM^j^_2.5_	–0.075
PM^k^_10_	–0.060
THC^l^	0.580^m^
Nitrogen dioxide	–0.314^g^
Ozone	0.030
Nitrogen oxides	–0.400^g^
Carbon monoxide	–0.412^m^
Methane	0.644^m^
Sulfur dioxide	–0.624^m^
Nitric oxide	–0.473^m^
NMHC^n^	0.595^m^

a AT: average temperature.

b ADT: average dew point temperature.

c GSR: global solar radiation.

d DP: days with precipitation.

e ACC: average cloud cover.

f AWS: average wind speed.

g Significant correlation at *P*<.05.

h ARH: average relative humidity.

i AWD: average wind direction.

j PM_2.5_: particulate matter with diameter ≤2.5 μm.

k PM_10_: particulate matter with diameter ≤10 μm.

l THC: total hydrocarbon.

m Significant correlation at *P*<.001.

n NMHC: nonmethane hydrocarbon.

### Rising Queries From GT for DED in Taiwan

Rising search queries from GT across different periods showing shifting interests are summarized in Table 2. In the pre–COVID-19 period, the focus was primarily on diagnostic and treatment methods such as intense pulsed light (IPL) and Schirmer test. During the COVID-19 lockdown, there was increased interest in natural remedies, such as omega-3 fatty acids, natural eye drops, and lutein. In the post–COVID-19 period, there were more frequent searches for self-diagnosis and treatment options such as dry eye self-diagnosis, forums, and sicca syndrome. The Chinese keywords that correspond to each English terms are listed in Table S5 in Multimedia Appendix 1.

The alignment between rising related queries from GT and accessible web-based information revealed a strong focus on DED symptoms, treatments, and self-care (Table S6 in Multimedia Appendix 1). The most frequently mentioned terms were *dry eye*, *dry eye symptoms*, and *dryness*, each appearing 80 times. Treatment-related queries were also prominent, such as *eye drops* (60 times), *artificial tears* (58 times), and *essential fatty acids* (34 times). Invasive treatments such as *IPL* (18 times) and *dry eye surgery* (22 times) were commonly mentioned. Other terms such as *contact lens* (53 times) and *Schirmer test* (23 times) were also popular. However, treatments such as *Chinese medicine* appeared only 5 times, and *cost of IPL* was not mentioned.

Before the COVID-19 pandemic, Taiwanese rising web searches about dry eye were split between understanding the condition (symptoms, causes, and prognosis) and new treatments such as IPL therapy and fish oil supplementation. During the COVID-19 lockdown, work-from-home screen fatigue and *mask-associated dry eye* likely increased discomfort, coinciding with reduced access to clinics [27,28]. Rising search behavior pivoted accordingly: people wanted some self-treated remedies, such as natural eye drops, vitamin A, omega-3, and fish oil. Once restrictions were eased, queries increasingly focused on documenting and professionalizing care. Terms such as self-tests, Schirmer values, *sicca syndrome ICD-10*, and *dry-eye treatment near me* indicate that patients were reengaging with ophthalmologists, insurance reimbursements, and telehealth workflows they had become familiar with during the pandemic. Together, the 3 phases trace a shift from curiosity, through pandemic-induced self-reliance, to post–COVID-19 integration of professional and community resources for chronic ocular surface disease.

**Table 2.** Top rising Google Trends queries for dry eye disease in Taiwan.

Period and ranking^a^	Queries	Rising
Pre–COVID-19 pandemic		
1	Intense pulsed light	Breakout^b^
2	Will dry eye disease get better	Breakout
3	Schirmer test	Breakout
4	Dry eye treatment	Breakout
5	Chalazion	Breakout
6	Omega-3	Breakout
7	Dry eye causes	110%
8	Dry eye symptoms	100%
9	What to eat for dry eye	90%
10	Will dry eye get better	70%
11	Fish oil	50%
COVID-19 pandemic		
1	Omega 3	Breakout
2	Natural eye drops	Breakout
3	Lutein	450%
4	Dry eye symptoms	170%
5	Intense pulsed light cost	170%
6	Dry eye improvement	110%
7	Conjunctivitis	80%
8	Vitamin A	80%
9	Fish oil	60%
10	Will dry eye get better	50%
Post–COVID-19 pandemic		
1	Dry eye self-diagnosis	Breakout
2	Dry eye forum	Breakout
3	Sicca syndrome ICD-10^c^	Breakout
4	Dry eye treatment near me	Breakout
5	Dry eye forum	250%
6	How long does dry eye take to get better	190%
7	Intense pulsed light	90%
8	What to eat for dry eye	60%

a The top rising rankings in the related queries for relative search volume during the respective periods are included.

b The ”breakout” label indicates a sharp increase in relative search volume, with popularity growing by >5000%.

c *ICD-10*: *International Classification of Diseases, Tenth Revision*.

### Quality and Readability Assessment of Web-Based Information

After duplicate removal, 80 unique websites providing information on DED were evaluated (Table S7 in Multimedia Appendix 1). The mean DISCERN score of these websites was 2.14 (SD 0.40). Websites scored higher on clarity of aims (item 1: mean 3.79, SD 0.44), achieving aims (item 2: mean 3.26, SD 0.65), and relevance (item 3: mean 4.64, SD 0.64). However, significant weaknesses were observed in several critical areas, such as clarity on information sources (item 4: mean 1.24, SD 0.58), date of information production (item 5: mean 1.60, SD 0.63), provision of additional support details (item 7: mean 1.15, SD 0.42), and addressing areas of uncertainty (item 8: mean 1.09, SD 0.36). Furthermore, information regarding treatment choices was inadequate, with low scores for discussing risks of treatment (item 11: mean 1.71, SD 0.51), what happens if no treatment is used (item 12: mean 1.05, SD 0.31), impact on quality of life (item 13: mean 1.11, SD 0.39), presenting multiple treatment choices (item 14: mean 1.96, SD 0.60), and supporting shared decision-making (item 15: mean 1.11, SD 0.39). The mean overall quality rating (item 16) was also low at 2.23 (SD 0.62). Readability, assessed using the specified web-based tool, had a mean score of 21.13 (SD 4.51), corresponding to a level above high school.

## Discussion

### Principal Findings

Current studies exploring public interest for DED using GT data are limited [10,29]. In our study, we observed trends in RSV for DED and examined correlations with environmental parameters. We also identified public concerns and gaps in available medical information from Google search regarding DED–related queries.

Although the topic presented in GT was *DED*, we used the term DED interchangeably to align with terminology commonly used in previous studies [30-32]. The term *DED* is retained when specifically referring to the GT topic under analysis to accurately reflect the platform’s terminology. Our findings indicated that Taiwan has the highest RSV for DED globally according to GT data, which corresponded to the higher prevalence of DED in Taiwan compared to the worldwide average [33,34]. The rising RSV and higher prevalence for DED in Taiwan may be attributed to several factors, including increasing environmental pollution, the widespread use of digital devices, and cultural habits such as extensive air conditioner use [4,18,35,36]. The higher prevalence of DED in Taiwan compared to Western countries emphasized the impact of environmental and lifestyle differences between these regions [4,5,36]. For comparison, glaucoma in GT demonstrated no statistically significant trend, suggesting that the significant rise in DED-related RSV is less likely to result from unmeasured confounding factors and instead reflects a significant increase in public interest. This supports the robustness of the observed increase in RSV for DED.

Our study using the Kruskal-Wallis test found no significant monthly differences in RSV for DED in Taiwan. Previous research on seasonal variation in DED had mixed findings, with seasonal variations observed in high-altitude regions but not in low-altitude regions [18,37,38]. In high-altitude regions, the most severe DED symptoms, including discomfort, burning, and dryness, were observed, with the highest prevalence in spring and the lowest in summer [37,38]. One systematic review in China also revealed that the seasonal trend in DED was more evident in the northern regions than in the southern regions [39]. Taiwan’s low latitude results in relatively mild seasonal temperature changes, and its consistently rainy weather maintains high humidity throughout the year. These factors may contribute to the lack of seasonal variation in DED [18] and could also indirectly influence RSV for DED.

Environmental factors play a crucial role in the development and exacerbation of DED in Taiwan [18]. Previous studies have identified pollutants such as PM₂.₅, NO₂, CO, SO₂, and O₃ as contributing to ocular surface inflammation and DED symptoms [18-21]. For example, SO₂ and CO are associated with increased DED by potentially altering the structural composition of the outermost lipid layer of the precorneal tear film, leading to ocular discomfort and inflammation [40]. Concurrently, NO₂ is understood to disrupt the tear film lipid layer by altering the composition of O-acyl-ω-hydroxy fatty acids [41]. In our study, the Spearman correlation analysis revealed that higher concentrations of NO, NO₂, SO_2_, NOx, and CO were significantly associated with increased RSV, consistent with the pollutants identified in previous studies [18-21]. Moreover, in our study, we identified new air pollutants that may contribute to the occurrence of DED. These include CH_4_, THC, and NMHC, all of which demonstrated a strong correlation with RSV (*P*<.001). The findings reveal a significant and previously unrecognized association between hydrocarbon levels and public interest in DED, suggesting their potential role as emerging environmental risk factors for the disease. Hydrocarbons, including CH_4_, THC, and NMHC, are implicated in ocular surface pathology through several potential mechanisms. First, they contribute to the generation of potent secondary irritants, such as formaldehyde and acrolein, via photochemical reactions with NOₓ in the atmosphere [42,43]. These aldehydes are known to damage the corneal epithelium and conjunctiva directly [17]. Second, the reactive aldehydes may destabilize the tear film lipid layer, thereby increasing tear evaporation and inducing hyperosmolarity, a key driver in DED pathogenesis [44]. Furthermore, some studies suggest that exposure to hydrocarbons may correlate with systemic inflammatory markers, which could amplify ocular surface immune responses [45]. The symptoms of DED might prompt patients to seek information online, consequently increasing RSV. Future investigation is needed to elucidate the specific biological mechanisms through which hydrocarbons may trigger or exacerbate DED symptoms.

To further clarify the temporal alignment between RSV and the environmental parameters significantly correlated in the Spearman correlation analysis, we used DTW analysis [46,47]. Our findings revealed that CH₄ and THC had the smallest DTW distances with RSV, indicating a strong temporal correlation. The strong correlation between RSV and hydrocarbons like CH₄ and THC suggests that these pollutants may significantly impact public interest in DED. These results, combined with the strong correlations observed in the Spearman correlation analysis, suggest that hydrocarbons may play a significant role in influencing public interest in DED. Hydrocarbons are emitted from various sources, including vehicles, industrial processes, and agricultural activities. Exposure to high levels of hydrocarbons may cause ocular irritation, tear film instability, and inflammation of the ocular surface, leading to potential DED symptoms [22,48]. This may prompt individuals to seek information online, contributing to our findings of increased RSV for DED.

Regarding public interest in DED, previous studies showed that public trends toward DED often involved treatment information and home remedies but lacked information about treatment risks and necessity [49]. A study by Yu et al [50] examined public trends toward DED based on the Baidu index, showing that treatment options, subjective symptoms, and associated ocular diseases such as conjunctivitis and keratitis are the most popular terms related to DED. Similar to these findings, our study (Table S7 in Multimedia Appendix 1) also observed significant public interest in treatment options and subjective symptoms. However, our analysis further revealed unique trends in diagnostic queries and information-seeking behaviors, such as *Schirmer test* and *dry eye self-diagnosis*. Overall, our findings emphasize a growing public engagement with DED that extends beyond basic treatment information to include diagnosis, etiology, and symptoms.

Our analysis of rising search queries revealed a shift in public interest over time. During the COVID-19 pandemic, the trend likely reflected a shift toward self-care options and preventive health practices when access to traditional health care services was limited. In the post–COVID-19 period, the trend moved toward self-diagnosis and treatment options. This indicated increased health literacy and patient autonomy in health management [51,52].

Our analysis of rising Google search queries and RSV revealed significant public interest in specific aspects of DED, with notable shifts over time (Tables S5 and S6 in Multimedia Appendix 1). For instance, while topics such as *Chinese medicine* and *cost of IPL* emerged as rising queries, they were lacking in available web-based resources. Furthermore, when we compared these demonstrated public interests, we identified a clear gap between public interests and the availability of corresponding online medical information. Beyond the gaps in topic coverage, our DISCERN analysis (Table S7 in Multimedia Appendix 1) further highlights the nature of the quality deficiencies. While the evaluated websites generally presented clear aims and relevant content (DISCERN items 1‐3), they demonstrated crucial deficiencies in several areas of reliability, such as poor transparency of sources (item 4) and outdated information (item 5). Moreover, the information provided on treatment choices was notably lacking, particularly in outlining risks (item 11) and facilitating shared decision-making (item 15), culminating in a low overall quality score (item 16). These specific shortcomings, combined with the high readability level, suggest that much of the online DED information in Taiwan may not only be difficult to understand but also lacks the balance and comprehensive details necessary for informed health decisions. These findings collectively accentuate the need for health care providers and public health organizations to address these identified gaps. Public health organizations should promote clear digital guidance on DED self-care, natural remedies, and IPL. Clinicians and health care providers should then use this guidance in patient consultations to address self-treatment trends, clearly articulate potential risks, and guide patients toward professional intervention, ensuring cost implications are also discussed. A key strategy for all involved is to proactively engage in digital health communication by developing and disseminating accessible, evidence-based content through their websites and social media.

### Limitations

Our study has several limitations. First, RSV data from GT reflect search interest rather than confirmed DED prevalence or specific unmet health needs. Search behavior can be influenced by factors unrelated to disease incidence, such as media coverage, public health campaigns, seasonality, or emerging news events, which were not controlled for in this analysis [51,52]. Consequently, an observed increase in search interest does not necessarily equate to a parallel rise in DED prevalence or severity. Although our falsification outcome (glaucoma) showed no upward trend, the aforementioned residual confounding factors cannot be excluded. Second, the analysis is limited to Taiwan, so the findings may not be generalizable to other regions, and other unmeasured factors may also influence DED symptoms and public interest. Third, the general increase in internet penetration and use over time could contribute to the overall rising trend in RSV, although the use of a falsification outcome (glaucoma) showing no significant trend lends support to the specificity of the DED finding. Moreover, the lack of direct mechanistic studies on the role of hydrocarbons in DED represents a main limitation of our study. Further research should explore the mechanisms by which hydrocarbons influence the ocular surface and contribute to DED pathogenesis.

### Conclusions

This study provides a comprehensive analysis of GT data, demonstrating a significant increase in public interest in DED in Taiwan from December 2018 to July 2024. Novel air pollutants, particularly hydrocarbons such as CH_4_, THC, and NMHC, were identified as key factors strongly associated with public interest in DED. Our findings also emphasize the necessity for health education to bridge the gap between public interest and available public information.

## Supplementary material

10.2196/74317 Multimedia Appendix 1 Supplementary tables and figures.

## Abbreviations

CWA: Central Weather Administration
DED: dry eye disease
DTW: dynamic time warping
GT: Google Trends
IPL: intense pulsed light
MID: mean instantaneous derivative
NMHC: nonmethane hydrocarbon
PM₂.₅: particulate matter with diameter ≤2.5 μm
RSV: relative search volume
THC: total hydrocarbon
