# Supplementary material for: Public Interest in Dry Eye Disease and Its Association With Environmental Parameters in Taiwan: Google Trends Infodemiology Study
Source: JMIR Infodemiology. 2025 Nov 14;5:e74317. doi: 10.2196/74317 (PMC12617964; doi:10.2196/74317)
Supplement: Multimedia Appendix 1 [file infodemiology-v5-e74317-s001.docx]

**Supplementary Table 1.** Summary of Methodology and Key Findings for Dry Eye Disease

| **Methodological Component** | **Description** | **Key Findings** |
| --- | --- | --- |
| **Public Interest Trend Analysis** | **Temporal Trend:** Assessed monthly Relative Search Volume (RSV) for "Dry Eye Syndrome" (Dec 2018 - Jul 2024) using spline regression.  **Seasonal Variation:** Examined monthly RSV differences using the Kruskal-Wallis test. | A **statistically significant increasing trend** in RSV was found for Taiwan (Mean Instantaneous Derivative = 0.445, p < 0.001) and five of six special municipalities, with Kaohsiung showing borderline significance (p = 0.062). In contrast, the falsification outcome (glaucoma) showed no significant trend. No significant monthly or seasonal variation in RSV was detected. |
| **Environmental Association Analysis** | **Correlation:** Assessed the relationship between monthly RSV and various environmental parameters using Spearman's rank correlation.  **Temporal Alignment:** Measured the similarity between RSV and environmental time series using Dynamic Time Warping (DTW) to account for non-linear phase shifts. | **Significant Correlations:** Novel hydrocarbon pollutants (CH₄, THC, NMHC) and established pollutants (SO₂, CO, NO₂) showed significant correlations with RSV.  **Strongest Alignment:** Hydrocarbons (CH₄, THC) exhibited the smallest DTW distances with RSV, indicating the strongest temporal alignment. |
| **Analysis of Public Search Behavior** | **"Rising Queries" Analysis:** Extracted and categorized top rising search queries from three distinct periods (pre-COVID, during COVID, post-COVID) to identify shifts in public interest over time. | Public interest shifted from diagnosis & treatments (pre-COVID) to natural remedies & self-care (during COVID), and finally to self-diagnosis & professional care logistics (post-COVID). |
| **Online Information Assessment: Content & Gap Analysis** | Collected top 20 Google search results for 8 Chinese DED-related keywords (initial N=160), curated to a final set of 80 unique websites, and compared their content against public interest themes from "rising queries". | A significant content gap was identified between public interest and available information. High-interest rising queries such as "cost of IPL" and "Chinese medicine" were largely absent from the top search results (0% and 6.3% coverage, respectively). |
| **Online Information Assessment: Quality Assessment ^a^** | Quality and reliability of the 80 unique websites were independently evaluated by two ophthalmologists using the 16-item DISCERN instrument (1-5 scale). | Quality was generally "poor" with a mean DISCERN score of 2.14 ± 0.40. Websites scored particularly low on transparency of sources, discussion of treatment risks, and support for shared decision-making. |
| **Online Information Assessment: Readability Assessment ^b^** | Readability of the Chinese text was assessed using a validated online tool that provides a grade score. | The mean readability score corresponded to a college-level difficulty, far exceeding the recommended grade level for public health materials and suggesting the content is inaccessible to much of the general public. |

^a^ The topic coverage analysis compared popular public search queries against the information provided by these websites to identify gaps in content.

^b^ The readability score corresponds to a difficulty level; higher scores indicate that a higher level of education is required to comprehend the text.

**Supplementary Table 2.** Spline Regression for Slope of Interest Across Six Special Municipalities

in Taiwan

|  | Adjusted R² ^a, b^ | p-value  (Spline Regression) | Mean Instantaneous Derivative  (95% CI) ^b^ | p-value  (Bootstrap  Analysis) ^c^ |
| --- | --- | --- | --- | --- |
| Taiwan | 0.619 | *1.881x10^-13^* | 0.445 (0.308, 0.593) | *<0.001* |
| Taipei City | 0.598 | *1.019x10^-12^* | 0.501 (0.326, 0.663) | *<0.001* |
| New Taipei City | 0.478 | *3.165x10^-09^* | 0.437 (0.261, 0.621) | *<0.001* |
| Taoyuan City | 0.228 | *3.953x10^-04^* | 0.435 (0.102, 0.744) | *0.014* |
| Taichung City | 0.323 | *8.330x10^-06^* | 0.344 (0.150, 0.529) | *0.002* |
| Tainan City | 0.275 | *6.422x10^-05^* | 0.421 (0.110, 0.758) | *0.014* |
| Kaohsiung City | 0.351 | *2.305x10^-06^* | 0.232 (-0.016, 0.472) | 0.062 |

^a^ Data are presented as adjusted R² values of spline regression

^b^ Mean Instantaneous Derivative indicates the average monthly change in RSV for Dry Eye Syndrome over the study period.

^c^ The numbers in italics denote that the association is statistically significant (p < 0.05).

**Supplementary Table 3.** Spearman’s Correlation Between RSV and Environmental Parameters across Taiwan and Six Special Municipalities in Taiwan

| Parameter | Correlation coefficient ^a^ | Parameter | Correlation coefficient ^a^ | Parameter | Correlation coefficient ^a^ |
| --- | --- | --- | --- | --- | --- |
| Taipei | | | | | |
| AT | 0.032 | THC | 0.580** | NOx | -0.400* |
| ADT | 0.082 | ACC | -0.071 | AWD | 0.123 |
| EP | 0.107 | NO_2_ | -0.314* | CO | -0.412** |
| GSR | 0.206 | O_3_ | 0.030 | CH_4_ | 0.644** |
| PP | -0.038 | PM_10_ | -0.060 | SO_2_ | -0.624** |
| DP | -0.191 | AWS | -0.315* | NO | -0.473** |
| PM_2.5_ | -0.075 | ARH | 0.134 | NMHC | -0.595** |
| New Taipei City | | | | | |
| AT | -0.024 | THC | 0.038 | NOx | -0.293* |
| ADT | 0.072 | ACC | 0.096 | AWD | 0.249* |
| EP | 0.129 | NO_2_ | -0.313* | CO | -0.213 |
| GSR | 0.184 | O_3_ | 0.099 | CH_4_ | 0.398* |
| PP | 0.076 | PM_10_ | -0.149 | SO_2_ | -0.599** |
| DP | -0.044 | AWS | -0.221 | NO | -0.223 |
| PM_2.5_ | -0.119 | ARH | 0.298* | NMHC | -0.560** |
| Taoyuan | | | | | |
| AT | 0.132 | THC | 0.105 | NOx | -0.162 |
| ADT | 0.171 | ACC | -0.084 | AWD | 0.105 |
| EP | 0.184 | NO_2_ | -0.108 | CO | -0.171 |
| GSR | 0.231 | O_3_ | 0.215 | CH_4_ | 0.300* |
| PP | -0.073 | PM_10_ | 0.083 | SO_2_ | -0.406* |
| DP | -0.146 | AWS | -0.226 | NO | -0.313* |
| PM_2.5_ | 0.027 | ARH | 0.070 | NMHC | -0.384* |
| Taichung | | | | | |
| AT | -0.153 | THC | 0.627** | NOx | 0.008 |
| ADT | -0.165 | ACC | -0.069 | AWD | 0.224 |
| EP | -0.098 | NO_2_ | 0.036 | CO | 0.051 |
| GSR | -0.079 | O_3_ | 0.319* | CH_4_ | 0.683** |
| PP | -0.049 | PM_10_ | 0.149 | SO_2_ | -0.331* |
| DP | -0.116 | AWS | -0.055 | NO | -0.157 |
| PM_2.5_ | 0.019 | ARH | -0.204 | NMHC | -0.123 |
| Tainan | | | | | |
| AT | 0.197 | THC | 0.082 | NOx | -0.293* |
| ADT | 0.200 | ACC | 0.048 | AWD | -0.293* |
| EP | None | NO_2_ | -0.255* | CO | -0.319* |
| GSR | 0.392* | O_3_ | 0.014 | CH_4_ | 0.216 |
| PP | 0.021 | PM_10_ | -0.177 | SO_2_ | -0.476** |
| DP | 0.026 | AWS | -0.293* | NO | -0.497** |
| PM_2.5_ | -0.201 | ARH | -0.052 | NMHC | -0.296* |
| Kaohsiung | | | | | |
| AT | -0.251* | THC | 0.121 | NOx | -0.169 |
| ADT | -0.178 | ACC | -0.134 | AWD | -0.010 |
| EP | 0.020 | NO_2_ | -0.057 | CO | -0.201 |
| GSR | 0.108 | O_3_ | 0.174 | CH_4_ | 0.320* |
| PP | -0.249* | PM_10_ | 0.026 | SO_2_ | -0.385* |
| DP | -0.249* | AWS | -0.537** | NO | -0.443** |
| PM_2.5_ | 0.045 | ARH | 0.122 | NMHC | -0.360* |
| Taiwan | | | | | |
| AT | 0.037 | THC | 0.388* | NOx | -0.316* |
| ADT | 0.071 | ACC | -0.012 | AWD | -0.170 |
| EP | 0.120 | NO_2_ | -0.244* | CO | -0.299* |
| GSR | 0.210 | O_3_ | 0.215 | CH_4_ | 0.629** |
| PP | -0.041 | PM_10_ | -0.094 | SO_2_ | -0.631** |
| DP | -0.133 | AWS | -0.343* | NO | -0.514** |
| PM_2.5_ | -0.121 | ARH | 0.144 | NMHC | -0.595** |

^a^ Significant correlations (p < 0.05) are marked as one asterisk, and significant correlations (p < 0.001) are marked as two asterisks.

**Supplementary Table 4.** Data of DTW Distance between RSV and Environmental Parameters across Six Special Municipalities in Taiwan ^a^

| Variable | DTW Distance ^a^ | Variable | DTW Distance ^a^ | Variable | DTW Distance ^a^ |
| --- | --- | --- | --- | --- | --- |
| Taipei | | | | | |
| CH_4_ | 36.156 | AWD | 54.059 | AWS | 70.285 |
| THC | 39.101 | O_3_ | 56.774 | NO_2_ | 74.703 |
| ADT | 43.430 | DP | 57.045 | NOx | 77.201 |
| AT | 47.854 | ARH | 57.652 | NO | 80.213 |
| GSR | 47.906 | PP | 58.145 | CO | 82.597 |
| ACC | 49.988 | PM_2.5_ | 60.296 | NMHC | 94.231 |
| EP | 50.370 | PM_10_ | 69.491 | SO_2_ | 97.515 |
| New Taipei City | | | | | |
| CH_4_ | 43.718 | THC | 53.220 | CO | 66.567 |
| ARH | 45.989 | AWD | 53.264 | PM_10_ | 67.588 |
| GSR | 47.601 | PP | 53.741 | AWS | 70.275 |
| ADT | 49.313 | O_3_ | 54.824 | NOx | 70.548 |
| AT | 50.108 | ACC | 56.234 | NO | 73.324 |
| DP | 52.168 | PM_2.5_ | 63.573 | NMHC | 82.405 |
| EP | 52.184 | NO_2_ | 66.321 | SO_2_ | 108.453 |
| Taoyuan | | | | | |
| CH_4_ | 49.044 | THC | 56.074 | AWD | 59.608 |
| GSR | 53.414 | O_3_ | 56.287 | NOx | 61.154 |
| AT | 53.436 | PM_2.5_ | 57.134 | DP | 62.211 |
| ADT | 54.216 | ACC | 57.239 | CO | 63.023 |
| ARH | 55.462 | AWS | 58.143 | NO | 65.489 |
| PP | 55.710 | PM_10_ | 58.496 | NMHC | 66.424 |
| EP | 55.944 | NO_2_ | 58.605 | SO_2_ | 79.771 |
| Taichung | | | | | |
| THC | 45.009 | AWS | 54.057 | CO | 61.513 |
| CH_4_ | 46.485 | DP | 54.725 | NOx | 62.831 |
| AT | 47.474 | GSR | 55.572 | NO_2_ | 63.992 |
| ADT | 49.388 | PM_10_ | 56.260 | NO | 64.473 |
| O_3_ | 52.678 | ARH | 57.484 | AWD | 64.995 |
| ACC | 53.046 | PP | 58.088 | NMHC | 67.491 |
| EP | 53.664 | PM_2.5_ | 60.371 | SO_2_ | 71.132 |
| Tainan | | | | | |
| THC | 46.719 | AT | 55.575 | PM_2.5_ | 62.789 |
| CH_4_ | 46.921 | AWD | 57.029 | NO_2_ | 65.573 |
| ADT | 53.795 | NO | 57.054 | PM_10_ | 66.051 |
| GSR | 54.347 | DP | 58.186 | CO | 66.100 |
| ARH | 54.755 | PP | 58.341 | NMHC | 66.630 |
| ACC | 55.448 | O_3_ | 58.555 | SO_2_ | 76.666 |
| AWS | 55.468 | NOx | 62.530 |  |  |
| Kaohsiung | | | | | |
| CH_4_ | 50.242 | THC | 57.101 | NO_2_ | 65.742 |
| ARH | 51.298 | PM_10_ | 57.755 | CO | 65.791 |
| ADT | 53.145 | EP | 58.036 | NMHC | 66.770 |
| AT | 54.996 | ACC | 59.209 | NOx | 67.023 |
| O_3_ | 55.607 | DP | 60.480 | AWS | 71.379 |
| PM_2.5_ | 56.261 | AWD | 64.437 | SO_2_ | 73.297 |
| GSR | 57.051 | PP | 64.453 | NO | 74.493 |

^a^ Lower DTW distances indicate stronger temporal alignment between RSV and the environmental parameters, supporting the observed correlations in the Spearman’s correlation analysis.

**Supplementary Table 5.** Categorized Rising Related Queries of Google Trends for Dry Eye Syndrome in Taiwan

| Related queries |  |  |  |  |
| --- | --- | --- | --- | --- |
| Symptoms | Treatment | Etiology, related diseases and clinical course | Diagnosis | Others |
| 1. **Dry eye** 2. 乾眼 3. 眼乾 4. 眼睛乾 5. Dry eye 6. Dry eyes 7. **Dry eye symptoms** 8. 乾眼症症狀 9. 乾燥 10. Dryness 11. **Dry eye syndrome** 12. 乾眼症 13. Sicca 14. Sicca syndrome 醫學 15. Sicca syndrome icd 10 | 1. **Eyedrops** 2. 乾眼症眼藥水 3. **Artificial tear**   (1) 人工淚液   1. **Lutein** 2. 葉黃素 3. 乾眼症葉黃素 4. **Essential fatty acid** 5. 魚油 6. 乾眼症魚油 7. Omega-3 8. **Intense pulse light (IPL)** 9. 脈衝光 10. 脈衝光乾眼症 11. **Dry eye surgery**   (1) 乾眼症手術   1. **Chinese medicine** 2. 乾眼症中醫 3. **Others** 4. Dry eyes treatment 5. 乾眼症治療 (dry eye treatment) 6. 乾眼症改善 (dry eye improvement) 7. 乾眼症痊癒 (dry eye cured) 8. 乾眼症怎麼辦 (how to do with dry eye) 9. 乾眼症吃什麼 (what to eat with dry eye) | 1. **Etiology**   (1) 乾眼症原因   1. **Contact lens**   (1) 隱形眼鏡   1. **Related diseases** 2. 結膜炎(conjunctivitis) 3. 霰粒腫 (chalazion) 4. **Clinical course** 5. 乾眼症會好嗎 6. 乾眼症多久會好 | 1. **Schirmer** 2. Schirmer   test   1. **Self-diagnosis** 2. 乾眼症自我檢測 | 1. **Ophthalmology** 2. 眼科 3. 眼科學 4. **Cost of IPL**   (1) 乾眼症脈衝光費用   1. **Dry eye forum** 2. **Dry eye treatment near me** |

^a^ The related topics are in bold, with the corresponding Chinese and English terms from Google Trends listed above.

**Supplementary Table 6.** Rising Related Queries for Dry Eye Syndrome in Taiwan and Their Frequency in Online Search Results ^a, b^

| Related queries | | | | |
| --- | --- | --- | --- | --- |
| Symptoms | Treatment | Etiology, related diseases and clinical course | Diagnosis | Others |
| 1. Dry eye: (80) 2. Dry eye symptoms: (80) 3. Dry eye syndrome: (80) | 1. Eyedrops: (60) 2. Artificial tear:   (58)   1. Essential fatty acid: (34) 2. Dry eye surgery: (22) 3. Intense pulse light (IPL): (18) 4. Lutein: (8) 5. Chinese medicine: (5) 6. Others: (8) to (66) | 1. Etiology: (65) 2. Contact lens: (53) 3. Related diseases: (20) 4. Clinical course: (15) | 1. Schirmer: (23) 2. Self-diagnosis: (17) | 1. Ophthalmology: (80) 2. Dry eye treatment near me: (43) 3. Dry eye forum: (18) 4. Cost of IPL: (0) |

^a^ These websites primarily contained health education platforms, media outlets, hospitals information, and clinics.
^b^ The frequency number in the table reflects how often each query appeared in the 80 websites, indicating its public popularity.

**Supplementary Table 7.** DISCERN Instrument Evaluation of Online Dry Eye Disease Information (n=80 Websites)

| DISCERN Item No. | DISCERN Question/Criterion | Mean Score (± SD) ^a, b^ |
| --- | --- | --- |
| Section 1: Reliability of the Publication | | |
| 1 | Are the aims clear? | 3.79±0.44 |
| 2 | Does it achieve its aims? | 3.26±0.65 |
| 3 | Is it relevant? | 4.64±0.64 |
| 4 | Is it clear what sources of information were used to compile the publication (other than the author or producer)? | 1.24±0.58 |
| 5 | Is it clear when the information used or reported in the publication was produced? | 1.60±0.63 |
| 6 | Is it balanced and unbiased? | 2.95±0.73 |
| 7 | Does it provide details of additional sources of support and information? | 1.15±0.42 |
| 8 | Does it refer to areas of uncertainty? | 1.09±0.36 |
| Section 2: Quality of Information on Treatment Choices | | |
| 9 | Does it describe how each treatment works? | 2.71±0.48 |
| 10 | Does it describe the benefits of each treatment? | 2.70±0.49 |
| 11 | Does it describe the risks of each treatment? | 1.71±0.51 |
| 12 | Does it describe what would happen if no treatment is used? | 1.05±0.31 |
| 13 | Does it describe how the treatment choices affect overall quality of life? | 1.11±0.39 |
| 14 | Is it clear that there may be more than one possible treatment choice? | 1.96±0.60 |
| 15 | Does it provide support for shared decision-making? | 1.11±0.39 |
| Section 3: Overall Rating | | |
| 16 | What is the overall quality of the publication as a source of information about treatment choices? | 2.23±0.62 |
|  | Overall Average Score per Question | 2.14±0.40 |

^a^ Scoring scale: 1 = Criterion definitively not met (major shortcomings); 2 = Criterion not met (some shortcomings); 3 = Criterion partially met (unclear or aspects missing); 4 = Criterion met (minor shortcomings); 5 = Criterion definitively met (minimal shortcomings).

^b^ The "Overall Average Score per Question" is the mean of the average scores obtained for each of the 16 questions across all 80 websites, as reported in the main manuscript. Individual question means (±SD) provide a more granular view of specific strengths and weaknesses.


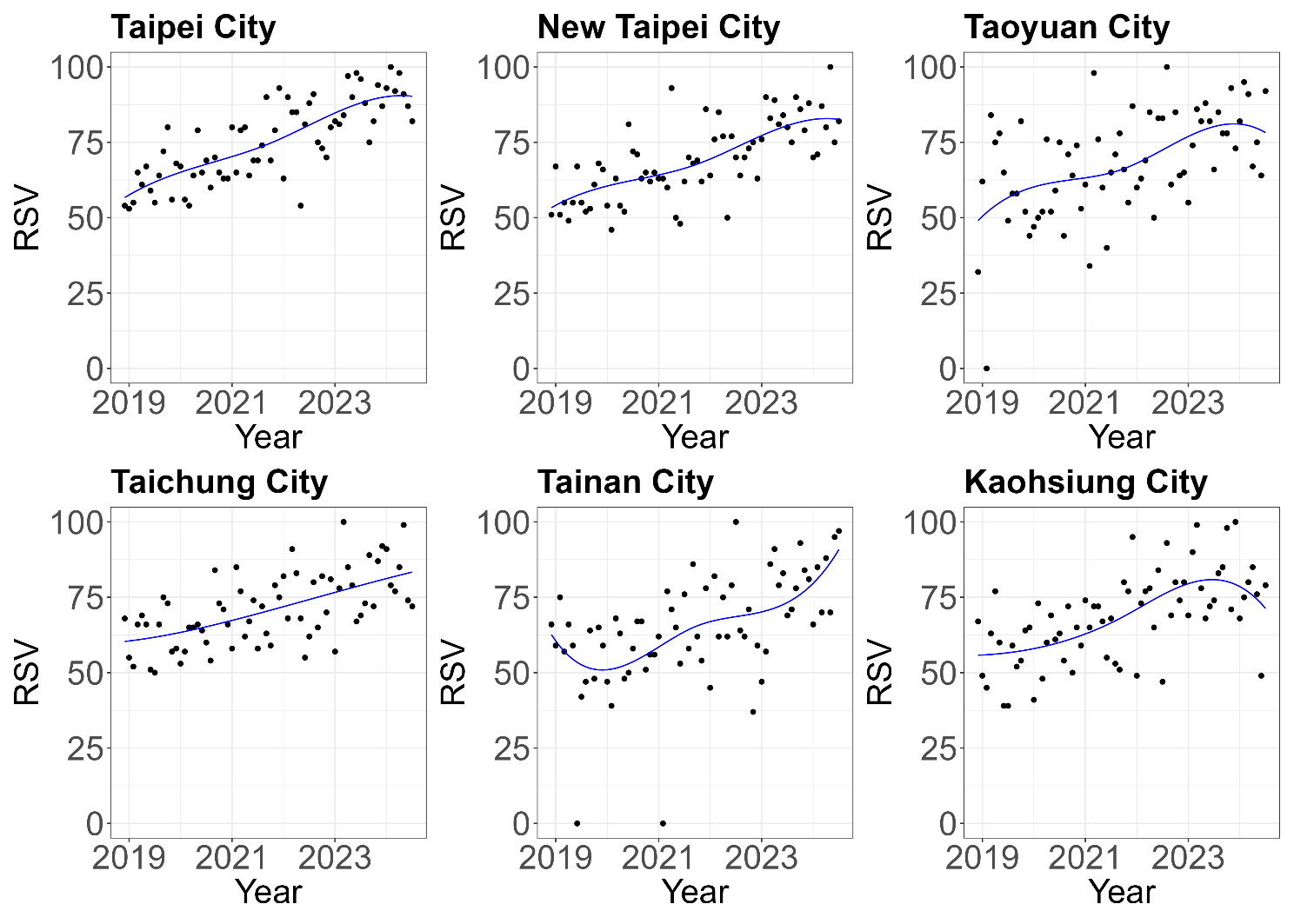


**Supplementary Figure 1.** **The Spline Regression of Relative Search Volume (RSV) for Dry Eye Syndrome in Six Special Municipalities in Taiwan**

The figure presents the spline regression model for the RSV for dry eye syndrome across six special municipalities in Taiwan from December 2018 to July 2024. The spline regression curves demonstrate significant positive trends in most cities (p < 0.05), with Kaohsiung revealing borderline significance (p = 0.062).


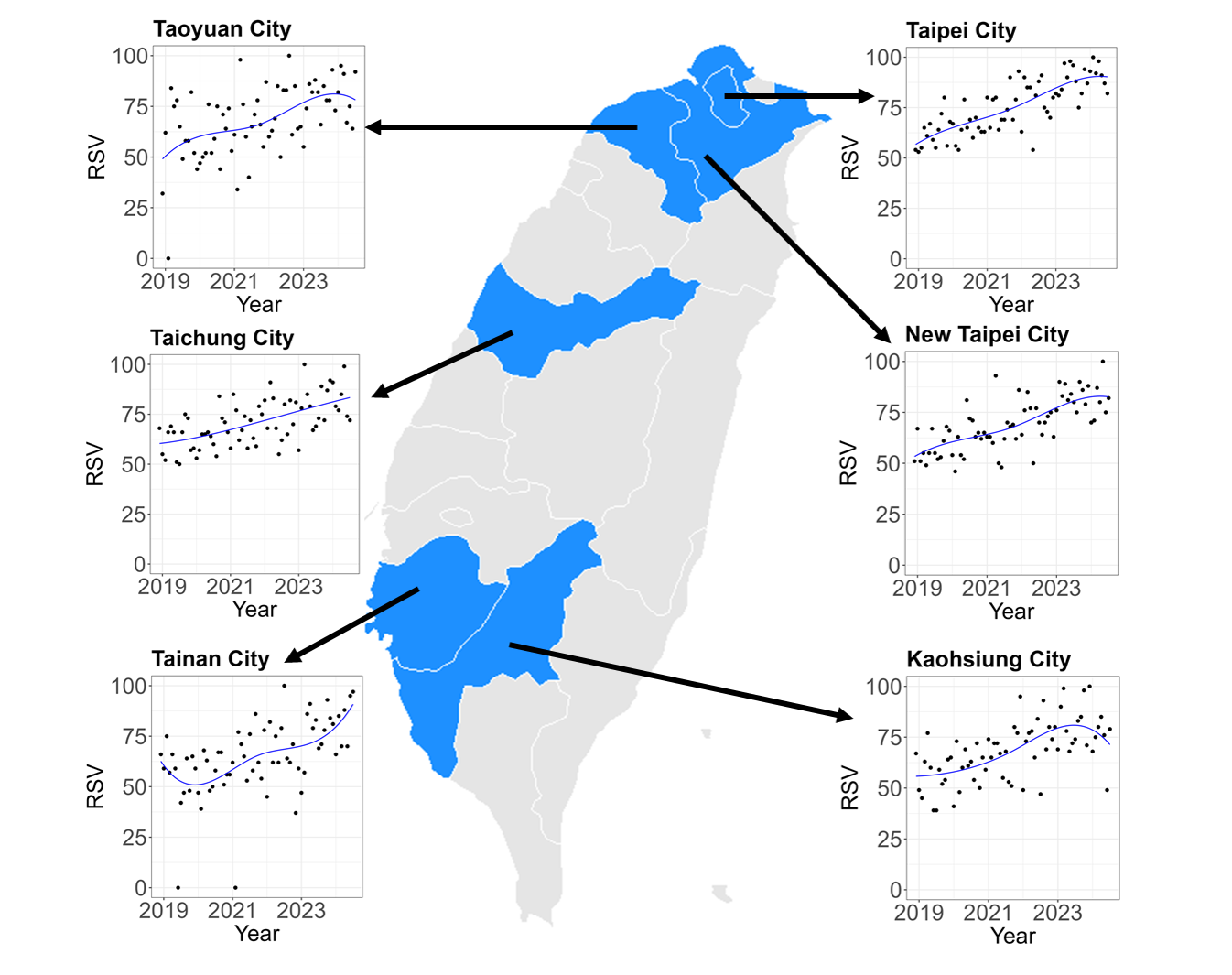


**Supplementary Figure** **2. Geographical Distribution and Temporal Trends of Public Interest in Dry Eye Syndrome across Taiwan's Six Special Municipalities**

The figure presents a map of Taiwan with its six special municipalities included in the study. Each inset plot displays the monthly RSV for dry eye syndrome from December 2018 to July 2024 for the corresponding municipality. The blue line in each inset represents the spline regression curve, visually demonstrating the increasing trend in public search interest over the study period for each region.


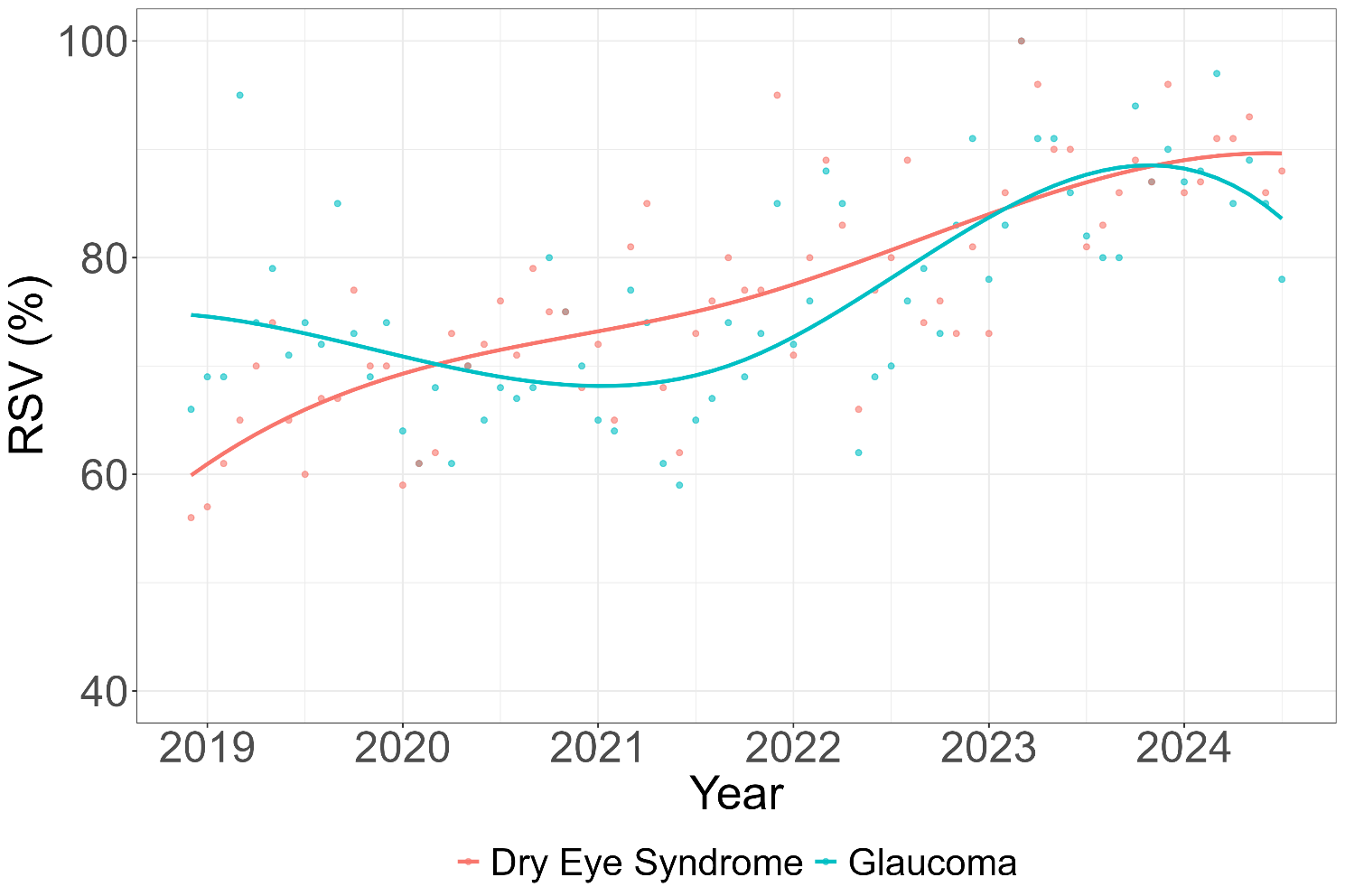


**Supplementary Figure 3. Combined Trend in Relative Search Volume (RSV) for Glaucoma and Dry Eye Syndrome**

This figure illustrates the RSV trend for glaucoma and dry eye syndrome in Taiwan, based on Google Trends data from December 2018 to July 2024. The red line represents the RSV for dry eye syndrome, with spline regression analysis revealing a mean instantaneous derivative (MID) of 0.445 and a statistically significant positive trend (95% CI: 0.308 to 0.593, p < 0.001). The blue line represents spline regression for glaucoma, where the MID of 0.120 suggests no statistically significant change (95% CI: -0.020 to 0.258, p = 0.118).
